# Does the primary route of spread have a prognostic significance in stage III non-serous epithelial ovarian cancer?

**DOI:** 10.1186/s13048-018-0393-0

**Published:** 2018-03-05

**Authors:** Hanifi Sahin, Mehmet Mutlu Meydanli, Mustafa Erkan Sari, Ibrahim Yalcin, Gonca Çoban, Nazlı Topfedaisi Ozkan, Zeliha Firat Cuylan, Baki Erdem, Kemal Gungorduk, Özgür Akbayir, Murat Dede, Mustafa Coşkun Salman, Tayfun Güngör, Ali Ayhan

**Affiliations:** 1 Department of Gynecologic Oncology, Zekai Tahir Burak Women’s Health Training and Research Hospital, Faculty of Medicine, University of Health Sciences, Ankara, Turkey; 2 Division of Gynecologic Oncology, Department of Obstetrics and Gynecology, Faculty of Medicine, Baskent University, Adana, Turkey; 3 Department of Gynecologic Oncology, Kanuni Sultan Suleyman Teaching and Research Hospital, Faculty of Medicine, University of Health Sciences, Istanbul, Turkey; 4 Department of Gynecologic Oncology, Tepecik Education and Research Hospital, Faculty of Medicine, University of Health Sciences, Izmir, Turkey; 5 Department of Obstetrics and Gynecology, Gulhane Training and Researh Hospital, Faculty of Medicine, University of Health Sciences, Ankara, Turkey; 6 Division of Gynecologic Oncology, Department of Obstetrics and Gynecology, Faculty of Medicine, Hacettepe University, Ankara, Turkey

**Keywords:** Clear cell adenocarcinoma, Endometrioid adenocarcinoma, Epithelial ovarian cancer, Lymph node dissection, Mucinous adenocarcinoma, Survival analysis

## Abstract

**Background:**

The purpose of this retrospective study was to determine the prognosis of non-serous epithelial ovarian cancer (EOC) patients with exclusively retroperitoneal lymph node (LN) metastases, and to compare the prognosis of these women to that of patients who had abdominal peritoneal involvement.

**Methods:**

A multicenter, retrospective department database review was performed to identify patients with stage III non-serous EOC at 7 gynecologic oncology centers in Turkey. Demographic, clinicopathological and survival data were collected. The patients were divided into three groups based on the initial sites of disease: 1) the retroperitoneal (RP) group included patients who had positive pelvic and /or para-aortic LNs only. 2) The intraperitoneal (IP) group included patients with > 2 cm IP dissemination outside of the pelvis. These patients all had a negative LN status, 3) The IP / RP group included patients with > 2 cm IP dissemination outside of the pelvis as well as positive LN status. Survival data were compared with regard to the groups.

**Results:**

We identified 179 women with stage III non-serous EOC who were treated at 7 participating centers during the study period. The median age of the patients was 53 years, and the median duration of follow-up was 39 months. There were 35 (19.6%) patients in the RP group, 72 (40.2%) in the IP group and 72 (40.2%) in the IP/RP group. The 5-year disease-free survival (DFS) rates for the RP, the IP, and IP/RP groups were 66.4%, 37.6%, and 25.5%, respectively (*p* = 0.002). The 5-year overall survival (OS) rate for the RP group was significantly longer when compared to those of the IP, and the IP/RP groups (74.4% vs. 54%, and 36%, respectively; *p* = 0.011). However, we were not able to define “RP only disease” as an independent prognostic factor for increased DFS or OS.

**Conclusions:**

Primary non-serous EOC patients with node-positive-only disease seem to have better survival when compared to those with extra-pelvic peritoneal involvement.

## Background

Epithelial ovarian cancer (EOC) is known to metastasize to the retroperitoneal lymph nodes (LNs) but only about 9% spread to the LNs in the absence of or with minimal spread to the peritoneal cavity [1, 2]. Previous studies indicate that these cases have a better prognosis than that of tumors with abdominal peritoneal involvement [3–10]. Although informative, previous studies were limited by heterogeneous patient samples with regard to the histological subtype [3–5, 7, 9] or residual disease (RD) status [3, 4, 6–8, 10]. Additionally, frequent use of cyclophosphamide in first-line chemotherapy regimens might have acted as a confounding factor in earlier studies [3, 4, 6, 7].

Today, it is well-known that ovarian cancer is not a single disease but rather a group of diseases - each with different morphology and biologic behavior [11]. At least five main types are currently distinguished: high-grade serous carcinoma (HGSC [70%]), endometrioid carcinoma (10%), clear-cell carcinoma (10%), mucinous carcinoma (3%), and low-grade serous carcinoma (LGSC [< 5%]) [12]. It is well-known that tumor histology influences the incidence of nodal disease [13, 14], chemo sensitivity [15], and prognosis [16]. Thus, distinct histopathological features might characterize tumor cells metastasizing through the lymphatic route compared to the trans-coelomic route.

However, previous studies which have investigated the subgroup of patients with exclusively retroperitoneal LN metastases generally analyzed all histologic types simultaneously [3, 4, 7, 9, 10] and included mostly patients with serous EOC [3–10]. Under the current perspective that the different histological subtypes in ovarian cancer probably represent different disease entities [17], we wondered whether the favorable prognosis for serous EOC characterized by node-positive-only disease [8] is also valid for patients with non-serous EOC such as endometrioid, clear-cell and mucinous subtypes. Attributed to the rarity of the condition, we designed this multicenter, collaborative study in order to shed some light on this issue. The purpose of this retrospective study was to determine the prognosis of non-serous EOC patients with exclusively retroperitoneal LN metastases, and to compare the prognoses of these women to those of patients who had abdominal peritoneal involvement.

## Methods

### Study design and eligibility

Medical records of women who underwent primary surgical treatment for EOC between January 2007 and December 2016 at seven gynecologic oncology centers from Turkey were retrospectively reviewed. The study protocol was approved by the Local Institutional Review Board (Zekai Tahir Burak Women’s Health Training and Research Hospital, Faculty of Medicine, University of Health Sciences, Ankara, Turkey, IRB Approval Number:10, Date: Nov 2nd, 2016). All patients provided an informed consent regarding research use of their medical information at admission.

The study population included women who had non-serous EOC (i.e., endometrioid, clear-cell, mucinous, and mixed subtypes) with histopathologically proven Stage III [18] disease. Women were included if they underwent primary surgical treatment including total hysterectomy plus bilateral salpingo-oopherectomy, with bilateral pelvic and para-aortic lymphadenectomy and other surgical procedures resulting in optimal debulking. All patients had to have RD of 1 cm or less in order to be eligible. Patients who were cytoreduced to greater than 1 cm of RD were excluded. Since this study focused only on women having non-serous EOC; women with HGSC, those with LGSC were excluded as well as patients having no lymphadenectomy. We also excluded patients who received neoadjuvant chemotherapy, women with synchronous malignancies, and those with incomplete medical records.

### Clinical information

Patient data were extracted from 7 institutions with maintained EOC databases. With the eligible cases, the following information was abstracted from medical records: demographic characteristics, preoperative serum cancer antigen 125 (CA 125) level, date and type of surgical procedure, presence or absence of ascites, the status of peritoneal cytology examination (negative, positive, or not performed), size of the primary tumor, bilaterality, size of residual tumor after surgery, stage of disease, time to recurrence, site of recurrence, length of follow-up and survival. Tumor characteristics were abstracted from original pathology reports. Data were collected from centers with an online standardized form.

Data on the extent of surgery included number of total LNs harvested, number of pelvic LNs removed, and number of para-aortic LNs removed and number of metastatic LNs. All operations were performed by gynecologic oncologists with intent to achieve optimal cytoreduction. Lymphadenectomy was performed after completion of other cytoreductive procedures. The decision to perform lymphadenectomy was determined by the surgeon’s discretion.

All surgical specimens were examined and interpreted by gynecologic pathologists. Non-serous EOC was diagnosed after examination of permanent sections. Architectural grading was defined by standard International Federation of Gynecology and Obstetrics (FIGO) criteria. All tumors were staged according to the 2014 FIGO staging system [18]. In patients treated before 2014, stage was determined retrospectively on the basis of surgical and pathologic assessment.

The current study investigated cases with mixed non-serous histologies (including mucinous, clear-cell, endometrioid, and transitional cell types) as a separate group, and did not assign mixed tumors according to the dominant component. Mixed tumors were diagnosed according to the World Health Organization (WHO) definition; in that more than one cell type was present, and the minority component accounted for at least 10% of the tumor. Mixed tumors containing serous component were excluded. For the purposes of this study, only pure tumors were classified as endometrioid, clear-cell or mucinous whereas tumors having more than one-cell type were classified as mixed.

The treatment policies were decided by the attending physician or by the multidisciplinary tumor board at each participating institution. Adjuvant chemotherapy was administered to all patients. The standard primary chemotherapy regimen included paclitaxel 175 mg/m^2^ plus carboplatin dosed at an area under curve of 5 or 6 every 21 days for 6 cycles.

Patients returned for follow-up evaluation every 3 months for the first 2 years, every 6 months for the next 3 years, and annually thereafter. Computed tomography or magnetic resonance imaging was performed annually. Survival data were last calculated on 31st December 2016. The survival status of the patients was determined as alive or dead at the time of the last follow-up. For all study subjects with a recorded death, this was confirmed by performing a social security death index search.

### Definitons

The patients were divided into three groups based on the initial sites of disease described by Rungruang et al. [9]: ***i)*** the retroperitoneal (RP) group included patients who had positive pelvic and/or para-aortic LNs only. By definition, these patients had < 2 cm intraperitoneal (IP) disease outside of the pelvis, ***ii)*** the intraperitoneal (IP) group included patients with > 2 cm IP dissemination outside of the pelvis. These patients all had a negative LN status, ***iii)*** the IP / RP group included patients with > 2 cm IP dissemination outside of the pelvis as well as positive LN status.

Optimal cytoreduction was defined as less than or equal to 1 cm maximal diameter of the largest residual tumor nodule at the completion of the primary operation. Maximal cytoreduction was defined as no macroscopic residual disease at the end of the primary operation. Suboptimal debulking was defined as > 1 cm of residual disease. Lymphadenectomy was defined as the performance of pelvic and para-aortic LN dissection at the same time. We defined pelvic lymphadenectomy as removal of the lymphatic tissue in the external, internal and common iliac and obturator regions. Para-aortic lymphadenectomy was defined as removal of the lymphatic tissue over the inferior vena cava and aorta beginning at the level of aortic bifurcation up to the left renal vessels.

Disease-free survival (DFS) was defined as the time, in months, from the date of primary surgery until the date of documented recurrence on the basis of clinical examination or radiologic imaging; or death from any cause, whichever occurred first, or the date of last contact for patients remaining alive without recurrent disease. Patients who had no active ovarian cancer at the last contact were censored in the DFS analysis. Overall survival (OS) was calculated as the time period, in months, between the date of primary surgery to the date of death or the last contact. Surviving patients were censored at their last known follow-up.

### Statistical analysis

Statistical analyses were performed using the statistical software package SPSS version 23.0 (SPSS, Inc., Chicago, IL). The data were expressed as median and range for continuous variables. The continuous variables such as age and tumor size have been divided into categories according to the median values. Binary variables were reported as counts and percentages.

One-way ANOVA was used to compare normally distributed variables among the groups (RP, IP, IP/RP). Levene test was used to assess the homogenity of variances. An overall *p*-value of less than 0.05 was considered to show a statistically significant result. When an overall significance was observed, pairwise post-hoc tests were performed using Tukey’s test. Kruskal-Wallis tests were conducted to compare the variables which were not distributed normally among the groups (RP, IP, IP/RP). The Mann-Whitney U test was performed to test the significance of pairwise differences using Bonferroni correction to adjust for multiple comparisons.

Survival curves were generated using the Kaplan-Meier method, and the differences between survival curves were calculated using the log-rank test. In order to evaluate the prognostic factors for DFS and OS, a univariate Cox-regression model was used. Any p-value of less than 0.05 in the univariate analysis was included into the multivariate analysis. A p-value < 0.05 was considered to indicate statistical significance.

## Results

During the study period, a total of 416 non-serous EOC were treated at seven participating centers. Of those, 276 women had histopathologically proven Stage III disease. Seventy two women who had suboptimal debulking and/or no lymphadenectomy were excluded from the study. We excluded 15 patients who received neoadjuvant chemotherapy, two with synchronous malignancies, and eight women with incomplete medical records. Therefore, the present analysis addresses the remaining 179 women with Stage III non-serous EOC.

The median age of the patients was 53 (range, 18-78) years, and the median duration of follow-up was 39 (range, 1-120) months. There were 35 (19.6%) patients in the RP group, 72 (40.2%) in the IP group and 72 (40.2%) in the IP/RP group. Table 1 demonstrates the clinical and pathological characteristics of women with regard to the route of spread. The groups were balanced for prognostic factors including age, menopausal status, histopathological subtype, FIGO grade, tumor size, and the number of LNs retrieved. Patients in the IP/RP group were more likely to have high baseline serum CA 125 levels (*p* = 0.001), ascites (*p* = 0.001), and bilateral tumors (*p* = 0.002) when compared to women in the RP and the IP groups. Women in the IP and the IP/RP groups were more likely to have positive peritoneal cytology (*p* < 0.001) when compared to women in the RP group (Table 1).

**Table 1:** Clinicopathologic characteristics of the patients according to the route of disease spread (*n* = 179)

	RP Group (*n* = 35)	IP Group (*n* = 72)	IP/RP Group (*n* = 72)	*p*
Age *(median, range)*	50 (18-74)	54 (28-78)	54 (27-77)	0.18
Menopausal Status *(n, %)*				0.23
Premenopausal	18 (51.4)	25 (34.7)	27 (37.5)	
Postmenopausal	17 (48.6)	47 (65.3)	45 (62.5)	
Histology *(n, %)*				0.42
Endometrioid	12 (34.3)	23 (31.9)	17 (23.6)	
Clear-cell	10 (28.6)	15 (20.8)	23 (31.9)	
Mucinous	10 (28.6)	24 (33.3)	13 (18.1)	
Mixed	3 (8.6)	10 (13.9)	19 (26.4)	
Grade^a^ (n, %)				0.078
1	5 (20)	8 (14)	6 (12.2)	
2	9 (36)	26 (45.6)	10 (20.4)	
3	11 (44)	23(40.4)	3 (67.3)	
Baseline Serum CA 125 (u/mL) (median, range)	118 (10-3305)	200 (11-9523)	400 (10-11,779)	**0.001** ^b^
Tumor size (cm) (median, range)	12 (4-30)	10 (3-39)	10 (3-40)	0.27
Ascites *(n, %)*				**0.001** ^b^
Yes	12 (34.3)	37 (51.4)	51 (70.8)	
No	23 (65.7)	35 (48.6)	21 (29.2)	
Peritoneal cytology *(n, %)*				**< 0.001** ^b^
Positive	12 (34.3)	53 (73.6)	53 (73.6)	
Negative	23 (65.7)	19 (26.4)	19 (26.4)	
Bilaterality				**0.002** ^b^
Yes	10 (28.6)	28 (38.9)	44 (61.1)	
No	25 (71.4)	44 (61.1)	28 (38.9)	
Total number of LNs removed (median, range)	43 (15-105)	37.5 (15-93)	40 (15-103)	0.71
Number of pelvic LNs removed (median, range)	25 (10-93)	28.5 (10-75)	28.5 (10-71)	0.97
Number of para-aortic LNs removed (median, range)	11 (5-46)	8.5 (5-45)	10 (5-54)	0.57
CRS				**0.009**
Optimal	17 (48.6)	17(30.9)	17(30.9)	
Maximal	18 (51.4)	55(69.1)	55(69.1)	

*Abbreviations: RP* retroperitoneal only disease, *IP* intraperitoneal only disease, *IP/RP* combination of intraperitoneal and retroperitoneal disease, *n* number, *LN* lymph node, *CRS* cytoreductive surgery

^a^Patients with clear-cell histology were excluded from the statistical analysis

^b^p values in bold characteristics refer to statistical significance

**Fig. 1:**
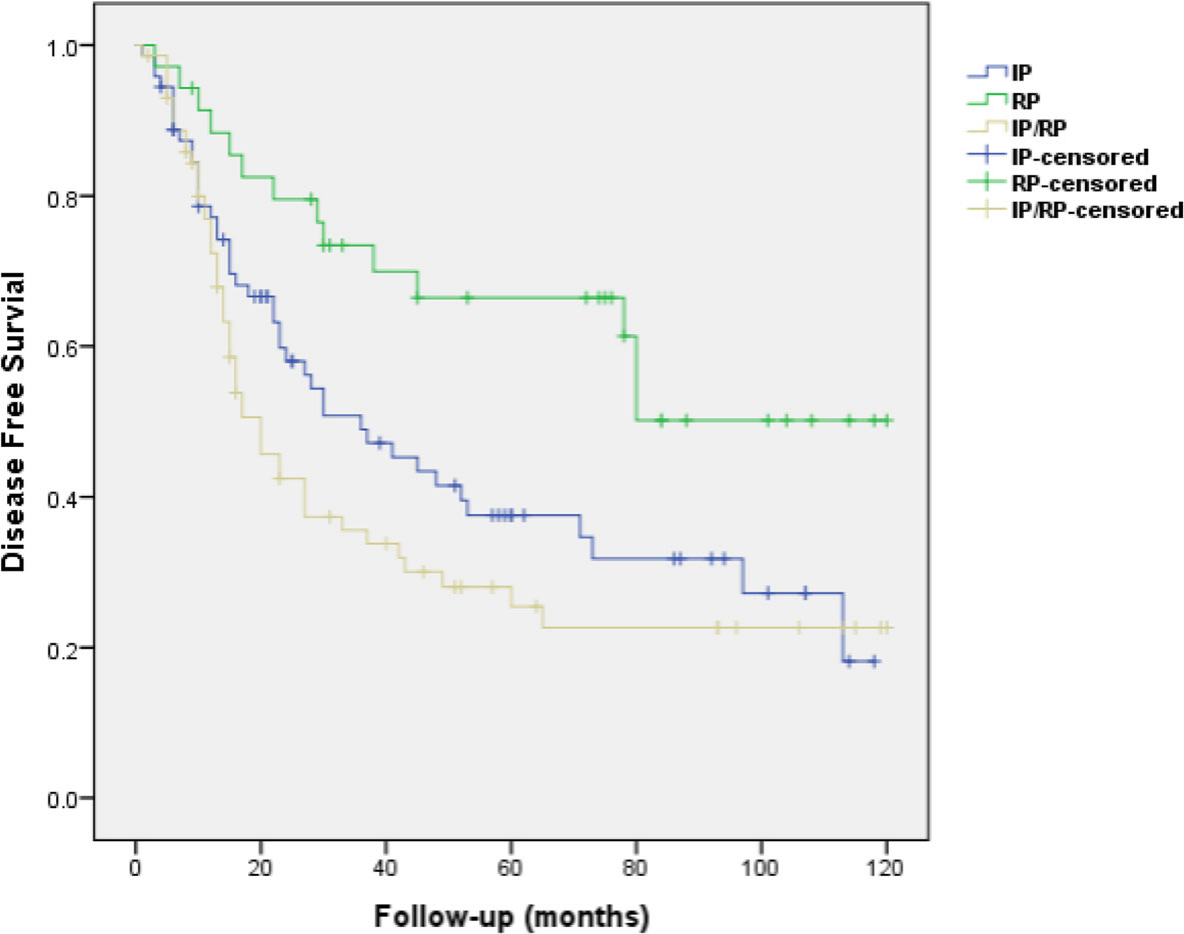
Disease-free survival curve of the retroperitoneal-only disease (RP) group (*n* = 35) compared to those of intraperitoneal only disease (IP) group (*n* = 72) and combination of intraperitoneal and retroperitoneal disease (IP/RP) group (*n* = 72) in women with non-serous epithelial ovarian cancer (*n* = 179)

The median DFS varied significantly among the groups. The median DFS for the RP group “has not been reached yet” compared to 36 months of median DFS in the IP group (%95 confidence interval [CI] 18.5-53.5 standard error [SE]: 8.92), and 20 months of median DFS in the IP/RP group (%95 CI 14.-26, SE: 3.06), *p* = 0.002 (Fig. 1). The 5-year DFS rates for the RP, the IP, and IP/RP groups were 66.4, 37.6 and 25.5%, respectively (*p* = 0.002). When DFS analyses were conducted by comparing the groups two at a time, the DFS rate for the RP group was significantly higher than that of the IP group (*p* = 0.011) as well as the IP/RP group (*p* = 0.001). However, there was no statistically significant difference in terms of DFS when the IP group was compared to the IP/RP group (*p* = 0.18).

**Fig. 2:**
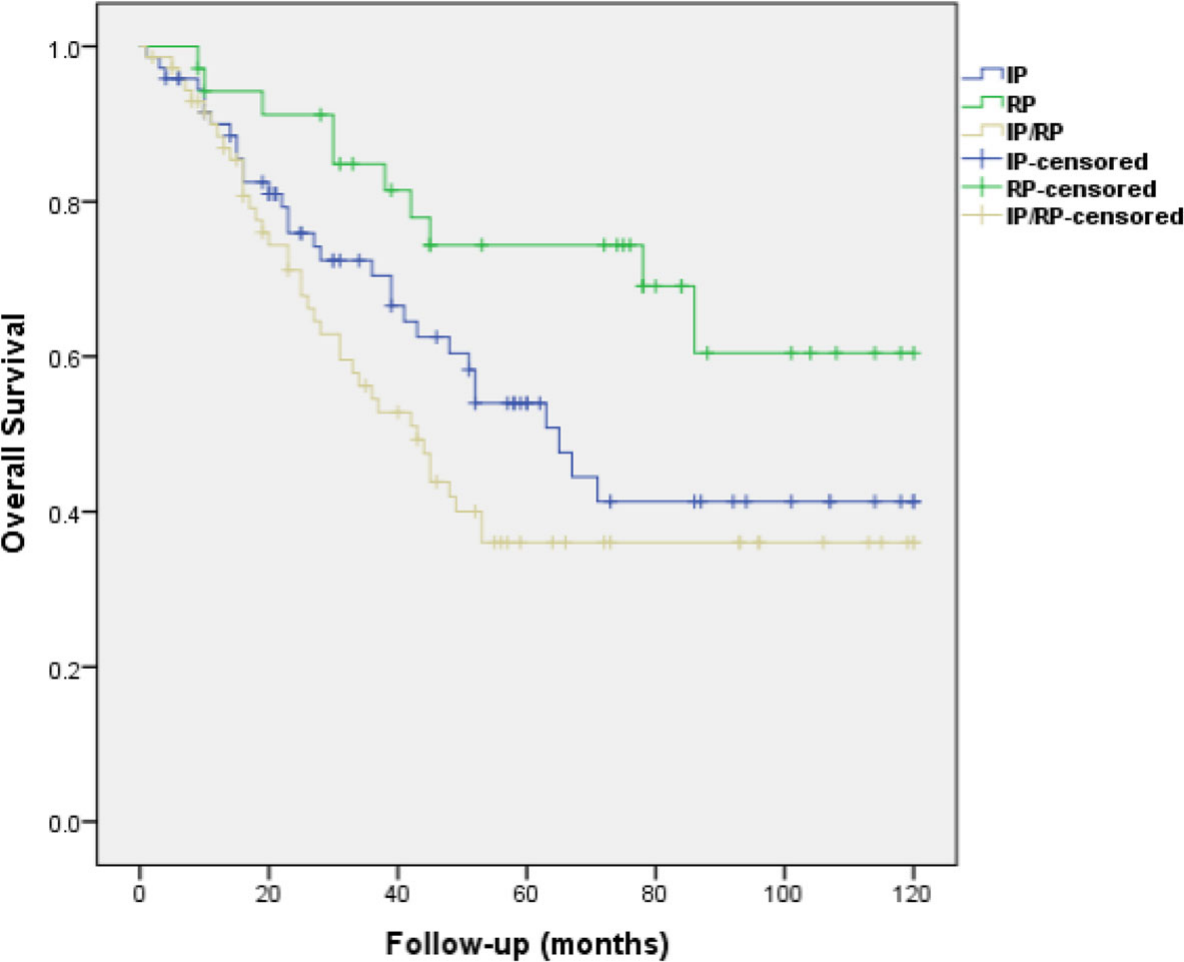
Overall survival curve of the retroperitoneal-only disease (RP) group (*n* = 35) compared to those of intraperitoneal only disease (IP) group (*n* = 72) and combination of intraperitoneal and retroperitoneal disease (IP/RP) group (*n* = 72) in women with non-serous epithelial ovarian cancer (*n* = 179)

The median OS of the RP group has not been reached yet. This figure was significantly longer than those of the IP (65 months, %95 CI 48.4-81.53, SE: 8.43), and the IP/RP (43 months, %95 CI 31.9-54, SE: 5.65) groups, *p* = 0.011 (Fig. 2). The 5-year OS rate for the RP group was significantly higher when compared to those of the IP, and the IP/RP groups (74.4% vs. 54%, and 36%, respectively; *p* = 0.011). When OS analyses were conducted by comparing the groups two at a time, the OS rate for the RP group was significantly higher than that of the IP group (*p* = 0.043) as well as the IP/RP group (*p* = 0.004). However, there was no statistically significant difference in terms of OS when the IP group was compared to the IP/RP group (*p* = 0.18).

For the entire cohort, univariate analysis revealed age > 53 years (*p* = 0.025), postmenopausal status (*p* = 0.03), IP/RP disease (*p* = 0.002), bilaterality (*p* = 0.01), omental involvement (*p* < 0.001), grade 3 disease (p < 0.001) and optimal debulking (p < 0.001) as significant factors for decreased DFS (Table 2). According to the multivariate analysis, grade 3 disease (Hazard ratio [HR] 2.3, 95%[CI] 1.42-3.92; *p* = 0.001), and optimal debulking (HR 5.3, 95% CI 2.37-12.14; p < 0.001), remained as independent prognostic factors for decreased DFS (Table 2).

**Table 2:** Univariate and multivariate analyses for disease-free survival in women with ovarian non-serous carcinoma

	DFS^a^	Events^b^	Univariate	Multivariate		
			*p*	HR	CI 95%	*p*
Age, y						
≤ 53	51.9%	44/87 (50.6%)				
> 53	26.7%	61/92 (66.3%)	**0.025**			
Menopausal Status						
Premenopausal	56.1%	32/70 (45.7%)	**0.003**			
Postmenopausal	27.7%	73/109 (66.9%)				
Histologic type						
Clear	29.3%	31/48 (64.6%)	0.310			
Müsinöz	48.3%	27/47 (53.8%)				
Endometrioid	41.8%	28/52 (53.8%)				
Mixt	33.6%	19/32 (59.3%)				
Route of Spread						
Intraperitoneal	37.6%	43/72 (59.7%)	**0.002**			
Retroperitoneal	66.4%	14/35 (40%)				
Intra+Retroperitoneal	25.5%	48/72 (66.6%)				
Peritoneal cytology						
Positive	38.1%	37/61 (60.6%)	0.717			
Negative	40.8%	68/118 (57.6%)				
Bilateralty						
Unilateral	49.4%	49/97 (50.5%)	**0.010**			
Bilateral	26.8%	56/82 (68.2%)				
Tumor size(cm)						
< 10	53.4%	21/45 (46.6%)	0.321			
≥ 10	63.3%	16/48 (33.3%)				
CA-125(IU/ml)						
< 250	38.4%	55/88 (31.4%)	0.720			
≥ 250	39%	47/84 (44.8%)				
Ascites						
Yes	40.6%	41/79 (51.9%)	0.131			
No	39%	64/100 (64%)				
LN involvement						
Yes	39.9%	62/107 (57.9%)	0.761			
No	37.6%	43/72 (59.7%)				
Omental involvement						
Yes	26.7%	73/106 (68.8%)	**< 0.001**			
No	57.5%	31/71 (43.6%)				
Grade						
Grade 1-2	63%	26/64 (40.6%)	**< 0.001**	**2.3**	**1.421-3.929**	**0.001**
Grade 3	24.4%	48/67 (71.6%)				
CRS						
Maximal	83.1%	9/51 (17.6%)	**< 0.001**	**5.3**	**2.374-12.142**	**< 0.001**
Optimal	23.3%	96/128 (75%)				

Characters in bold indicate statistical significance

*Abbreviations: DFS* disease free survival, *LN* lymph node, *CRS* cytoreductive surgery

^a^5-year recurrence free survival rate

^b^The number of cases with recurrence or death whichever occurred first

For the entire cohort, univariate analysis revealed age > 53 years (*p* = 0.004), postmenopausal status (*p* = 0.001), IP/RP disease (*p* = 0.011), omental involvement (*p* = 0.001), grade 3 disease (*p* = 0.003) and optimal debulking (p < 0.001) as significant factors for decreased OS (Table 3). Multivariate analysis demonstrated grade 3 disease (HR 1.90; 95% CI, 1.07-3.42, *p* = 0.028) and optimal debulking (HR 5.50; 95% CI, 1.96-15.62, *p* = 0.001) as independent predictors of decreased OS (Table 3).

**Table 3:** Univariate and multivariate analyses for overall survival in women with ovarian non-serous carcinoma

	OS^a^	Events^b^	Univariate	Multivariate		
			*p*	HR	CI 95%	*p*
Age, y						
≤53	61.5%	29/87 (33.3%)	**0.004**			
> 53	40.9%	51/92 (55.4%)				
Menopausal Status						
Premenopausal	66.4%	21/70 (30%)	**0.001**			
Postmenopausal	40.8%	59/109 (54.1%)				
Histologic type						
Clear	40.4%	26/48 (54.1%)	0.335			
Müsinöz	55.8%	23/47 (48.9%)				
Endometrioid	51.7%	21/52 (40.4%)				
Mixt	59.9%	10/32 (31.2%)				
Route of Spread						
Intraperitoneal	54%	31/72 (43%)	**0.011**			
Retroperitoneal	74.4%	10/35 (28.6%)				
Intra+Retroperitoneal	36%	39/72 (54.1%)				
Peritoneal cytology						
Positive	50.5%	51/118 (43.2%)	0.987			
Negative	51.1%	29/61 (47.5%)				
Bilateralty						
Unilateral	59.7%	40/97 (41.2%)	0.293			
Bilateral	40.2%	40/82 (48.7%)				
Tumor size(cm)						
< 10	47.7%	38/78 (48.7%)	0.169			
≥ 10	53.5%	42/101 (41.6%)				
CA-125(IU/ml)						
< 250	56.2%	39/88 (44.3%)	0.690			
≥ 250	44.5%	39/84 (46.4%)				
Ascites						
Yes	45.4%	50/100 (50%)	0.150			
No	58.1%	30/79 (37.9%)				
LN involvement						
Yes	49%	49/107 (45.8%)	0.955			
No	54%	31/72 (43%)				
Omental involvement						
Yes	38.9%	57/106 (53.7%)	**0.001**			
No	66.8%	22/71 (30.9%)				
Grade						
G1-2	69%	19/69 (27.5%)	**0.003**	**1.9**	**1.071-3.421**	**0.028**
G3	42%	35/67 (52.2%)				
CRS						
Maximal	94.5%	4/51 (7.8%)	**< 0.001**	**5.5**	**1.968-15.625**	**0.001**
Optimal	36.2%	76/128 (59.3%)				

Characters in bold indicate statistical significance

*Abbreviations: OS* overall survival, *LN* lymph node, *CRS* cytoreductive surgery

^a^5-year overall survival

^b^The number of cases with death

At the time of reporting, of 179 women with non-serous EOC, 99 (55.3%) were alive and 80 (44.7%) were dead. Since the number of women with node-positive-only disease was limited for each histotype group (i.e., endometrioid [*n* = 12], clear cell [*n* = 10], mucinous [*n* = 10], and mixed [*n* = 3]), we were unable to perform subgroup analyses for each histologic group.

## Discussion

To the best of our knowledge, this is the first study to investigate the prognostic impact of route of spread in advanced non-serous EOC with specific regard to the tumor histotype. Our results indicate a significant improvement in DFS and OS for RP only involvement compared to IP only tumor or a combination of IP and RP disease in a cohort of 179 women with non-serous EOC.

However, we should underline some limitations of the current study. First, the present study has potential limitations of selection bias and a relatively small number of patients in each histologic subgroup, which may be inherent in any retrospective study. Second, our study was restricted by the lack of central pathology review. However, given the low prevalence of node-positive-only disease in EOC, the study design used here was necessary to achieve a satisfactory sample size. Despite above limitations, our study contributes to the limited body of knowledge on this topic.

Accumulating evidences in the literature indicate that patients with exclusively LN metastases have an improved outcome than that of tumor with abdominal peritoneal involvement [3–10]. In some of the previous studies [5, 6, 10], the number of patients with non-serous EOC was not mentioned and no information has been given with regard to the histotype. However, in the study by Onda et al. [3], there were 48 patients with serous EOC whereas 55 patients had non-serous histology. Kanazawa et al. [4] reported on 54 patients with serous and 57 women with non-serous EOC. Baek et al. [7] included 197 serous and 65 non-serous EOC patients and reported on 41 patients with node-positive-only disease. In the largest study reported to date, Rungruang et al. [9] reported on 290 serous and 127 non-serous EOC patients. All of the above-mentioned studies were heterogenous with regard to the histotype.

The current study included 179 women with non-serous histotype with 35 patients with exclusively LN involvement. Our study represents the largest cohort of patients with non-serous EOC with exclusively LN involvement (Table 2). Compared with previous studies [3–7, 9, 10], our cohort seems to be more homogenous with all patients having non-serous histotype, and all patients having undergone optimal cytoreduction as well as LND. Additionally, all patients were treated with the standard paclitaxel/carboplatin regimen postoperatively. These factors seem to reduce the possibility of confounding, and enhance the reliability of the prognostic effects those have been estimated.

The primary routes of ovarian cancer metastasis include intra-peritoneal implantation of exfoliated cells at distinct sites and spreading through the retroperitoneal lymphatic channels [19]. The presence of tumor spreading mainly through lymphatic channels without intra-peritoneal dissemination suggests that such tumors might be associated with a favorable biologic behavior [10]. One plausible explanation for the favorable prognosis of those patients with retroperitoneal LN involvement only might be the higher optimal cytoreduction rate compared to the patients with intraperitoneal tumor implants > 2 cm [10]. However, given the significant differences in DFS and OS detected in our study among different patient groups according to the route of spread, we suggest that biology of the tumor rather than maximal surgical effort is associated with survival differences in these subgroups of patients as all patients included in the current study underwent optimal cytoreduction.

In a study of 107 patients with HGSC, Bakker et al. [8] reported that patients with pelvic and/or paraaortic LN metastases without extrapelvic peritoneal involvement (*n* = 13) had statistically significant better OS compared with other patients (*n* = 94) who had extrapelvic peritoneal involvement with or without nodal metastases. Our study confirmed these results in a homogenous population of non-serous EOC patients. However, Bakker et al. suggested that regional LN metastasis in ovarian serous carcinoma patients may be a poor prognostic indicator only in the presence of extra pelvic peritoneal metastasis [8]. We were not able to confirm this finding in our cohort as we were unable to demonstrate a significant difference in terms of DFS and OS when the IP group was compared to the IP/RP group.

We demonstrated a significant improvement in DFS and OS for node-positive-only disease compared to IP only tumor or a combination of IP and RP disease in a cohort of 179 women with non-serous EOC. Our results are comparable to those of Rungruang et al. [9] who included 203 patients with exclusively LN involvement. This is the largest study reported to date in terms of number of patients having node-positive-only disease [9]. However, it seems interesting that the number of patients with IP disease (*n* = 123) was less than the number of patients with only RP disease (*n* = 203) in the Rungruang study [9]; a finding not compatible with previous studies in the literature [3–8, 10]. It should be emphasized that non-serous histology constituted 30.5% of their study population. Table 4 demonstrates the number of patients according to the histotype in previous studies associated with node-positive- only disease in epithelial ovarian cancer.

**Table 4:** The number of patients according to the histotype in previous studies associated with node-positive- only disease in epithelial ovarian cancer

Author	Patients (*n*)	NP-OD^a^ (*n*)	Serous (*n*)	Endometrioid (*n*)	Clear-cell (*n*)	Mucinous (*n*)	Mixed (*n*)	Others (*n*)
Onda, 1998 [3]	103	14	48	9	22	17	2	5
Kanazawa, 1999 [4]	125	35	54	13	13	25	–	6
Cliby, 2006 [5]	NR	36	NR	NR	NR	NR	NR	NR
Ferrandina, 2007 [6]	118	26	NR	NR	NR	NR	NR	NR
Baek, 2008 [7]	272	41	197	23	4	13	23	12
Rungruang, 2012 [9]	417	203	290	37	34	9	29	18
Bakkar, 2014 [8]	107	13	107	–	–	–	–	–
Suh, 2014 [10]	483	33	NR	NR	NR	NR	NR	NR
Present study	179	35	–	52	48	47	32	–

*Abbreviation: NR* not reported

^a^Number of patients with *node positive-only disease*

Precise histopathologic diagnosis is mandatory for successful categorization and treatment of EOCs, as different histologic types respond differently to treatment [20]. We considered that it is crucial to investigate the prognosis of patients according to the pattern of spread with specific regard to histological subtypes in order to evaluate the outcome in individual subgroups in a multicenter analysis. However, the small number of patients in each histotype group did not allow us to perform subgroup analyses of patients with specific regard to the histotypes such as endometrioid, clear-cell, mucinous and mixed.

The strengths of the current study lie in its multicenter nature with a large number of patients with non-serous EOC. Our study has the advantage that its time period encompasses the last 10 years, during which all patients were treated with carboplatin plus paclitaxel with a uniformity of applying the same adjuvant treatment. Additionally, all of the patients in the current study underwent LND.

## Conclusions

We conclude that primary non-serous EOC patients with node-positive-only disease have better survival than those with extra-pelvic peritoneal involvement. Further studies with larger number of patients in each histologic subtype group are needed in order to demonstrate this survival benefit with specific regard to rare non-serous histotypes such as endometrioid, clear-cell, and mucinous carcinoma of the ovary.

## Abbreviations

CA 125: Cancer antigen 125
DFS: Disease-free survival
EOC: Epithelial ovarian cancer EOC
FIGO: International Federation of Gynecology and Obstetrics
HGSC: High-grade serous carcinoma
IP: Intraperitoneal
LGSC: Low-grade serous carcinoma
LN: Lymph node
OS: Overall survival
RD: Residual disease
RP: Retroperitoneal
WHO: World Health Organization

## References

[1] Berek JS (2009). Lymph node-positive stage IIIC ovarian cancer: a separate entity?. Int J Gynecol Cancer.

[2] Ayhan A, Gultekin M, Dursun P, Dogan NU, Aksan G, Guven S, Velipasaoglu M, Yuce K (2008). Metastatic lymph node number in epithelial ovarian carcinoma: does it have any clinical significance?. Gynecol Oncol.

[3] Onda T, Yoshikawa H, Yasugi T, Mishima M, Nakagawa S, Yamada M, Matsumoto K, Taketani Y (1998). Patients with ovarian carcinoma upstaged to stage III after systematic lymphadenctomy have similar survival to stage I/II patients and superior survival to other stage III patients. Cancer.

[4] Kanazawa K, Suzuki T, Tokashiki M (1999). The validity and significance of substage IIIC by node involvement in epithelial ovarian cancer: impact of nodal metastasis on patient survival. Gynecol Oncol.

[5] Cliby WA, Aletti GD, Wilson TO, Podratz KC (2006). Is it justified to classify patients to stage IIIC epithelial ovarian cancer based on nodal involvement only?. Gynecol Oncol.

[6] Ferrandina G, Scambia G, Legge F, Petrillo M, Salutari V (2007). Ovarian cancer patients with “node-positive-only” stage IIIC disease have a more favorable outcome than stage IIIA/B. Gynecol Oncol.

[7] Baek SJ, Park JY, Kim DY, Kim JH, Kim YM, Kim YT, Nam JH (2008). Stage IIIC epithelial ovarian cancer classified solely by lymph node metastasis has a more favorable prognosis than other types of stage IIIC epithelial ovarian cancer. J Gynecol Oncol.

[8] Bakkar R, Gershenson D, Fox P, Vu K, Zenali M, Silva E (2014). Stage IIIC ovarian/peritoneal serous carcinoma: a heterogeneous group of patients with different prognoses. Int J Gynecol Pathol.

[9] Rungruang B, Miller A, Richard SD, Hamilton CA, Rodriguez N, Bookman MA, Maxwell GL, Krivak TC, Horowitz NS (2012). Should stage IIIC ovarian cancer be further stratified by intraperitoneal vs. retroperitoneal only disease?: a gynecologic oncology group study. Gynecol Oncol.

[10] Suh DH, Kim TH, Kim JW, Kim SY, Kim HS, Lee TS, Chung HH, Kim YB, Park NH, Song YS (2013). Improvements to the FIGO staging for ovarian cancer: reconsideration of lymphatic spread and intraoperative tumor rupture. J Gynecol Oncol.

[11] Prat J (2012). Ovarian carcinomas: five distinct diseases with different origins, genetic alterations, and clinicopathological features. Virchows Arch.

[12] Kurman RJ, Carcangui ML, Herrington CS, Young RH (2014). Tumors of the ovary, WHO classification of tumours of female reproductive organs.

[13] Di Re F, Fontanelli R, Raspagliesi F, Di Re E (1989). Pelvic and Para-aortic lymphadenectomy in cancer of the ovary. Baillieres Clin Obstet Gynaecol.

[14] Petru E, Lahousen M, Tamussino K, Pickel H, Stranzl H, Stettner H, Winter R (1994). Lymphadenectomy in stage I ovarian cancer. Am J Obstet Gynecol.

[15] Sugiyama T, Kamura T, Kigawa J, Terakawa N, Kikuchi Y, Kita T, Suzuki M, Sato I, Taguchi K (2000). Clinical characteristics of clear cell carcinoma of the ovary: a distinct histologic type with poor prognosis and resistance to platinum-based chemotherapy. Cancer.

[16] Omura GA, Brady MF, Homesley HD, Yordan E, Major FJ, Buchsbaum HJ, Park RC (1991). Long-term follow-up and prognostic factor analysis in advanced ovarian carcinoma: the gynecologic oncology group experience. J Clin Oncol.

[17] Vaughan S, Coward JI, Bast RC, Berchuck A, Berek JS, Brenton JD, Coukos G, Crum CC, Drapkin R, Etemadmoghadam D (2011). Rethinking ovarian cancer: recommendations for improving outcomes. Nat Rev Cancer.

[18] Prat J, Oncology FCoG (2014). Staging classification for cancer of the ovary, fallopian tube, and peritoneum. Int J Gynaecol Obstet.

[19] Powless CA, Aletti GD, Bakkum-Gamez JN, Cliby WA (2011). Risk factors for lymph node metastasis in apparent early-stage epithelial ovarian cancer: implications for surgical staging. Gynecol Oncol.

[20] Mutch DG, Prat J (2014). 2014 FIGO staging for ovarian, fallopian tube and peritoneal cancer. Gynecol Oncol.

